# Impact of CYP2E1 Polymorphisms on Preoperative Paracetamol Analgesic Response in Patients with Lower Extremity Fractures

**DOI:** 10.3390/ph19060824

**Published:** 2026-05-25

**Authors:** Barış Kocabay, Nusret Ök, Sinem Yenil Kocabay, Zeynep Dündar Ök, Ali Çağdaş Yörükoğlu, Gergana Lengerova, Martina Bozhkova, Steliyan Petrov, Aylin Köseler

**Affiliations:** 1 Department of Orthopedics and Traumatology, Acıpayam State Hospital, 20160 Denizli, Türkiye; brskocabay94@gmail.com; 2 Department of Orthopedics and Traumatology, Faculty of Medicine, Pamukkale University, 20160 Denizli, Türkiye; acagdasy@pau.edu.tr; 3 Department of Orthopedic Rehabilitation, Faculty of Physiotherapy and Rehabilitation, Pamukkale University, 20160 Denizli, Türkiye; syenil@pau.edu.tr; 4 Department of Rheumatology, Denizli State Hospital, 20160 Denizli, Türkiye; zynpdundar@gmail.com; 5 Department of Medical Microbiology and Immunology “Prof. Dr. Elissay Yanev”, Medical University of Plovdiv, 4002 Plovdiv, Bulgaria; gergana.lengerova@mu-plovdiv.bg (G.L.); martina.bozhkova@mu-plovdiv.bg (M.B.); steliyan.petrov@mu-plovdiv.bg (S.P.); 6 Research Institute, Medical University of Plovdiv, 4002 Plovdiv, Bulgaria; 7 Center of Competence—Personalized Innovative Medicine, 4002 Plovdiv, Bulgaria; 8 Department of Biophysics, Faculty of Medicine, Pamukkale University, 20160 Denizli, Türkiye

**Keywords:** paracetamol, CYP2E1, pharmacogenetics, polymorphism, pain management, VAS, orthopedic

## Abstract

**Background**: Paracetamol is widely used for acute pain management in orthopedic trauma; however, interindividual variability in analgesic response remains insufficiently understood. Cytochrome P450 2E1 (CYP2E1), a key enzyme involved in paracetamol metabolism and the formation of the toxic metabolite N-acetyl-p-benzoquinone imine (NAPQI), may contribute to this variability. This study aimed to investigate the relationship between CYP2E1 gene polymorphisms and the analgesic efficacy of paracetamol in patients with lower extremity fractures. **Methods**: A total of 127 patients with lower extremity fractures and 100 healthy controls were included. All patients received 1000 mg of intravenous paracetamol. Pain intensity was assessed using the Visual Analog Scale (VAS) at baseline and at 30, 60, and 120 min after administration. Genotyping of CYP2E1 polymorphisms (*1A, *5B, *6, and *7B) was performed using PCR-RFLP. Differences in the VAS scores and analgesic response were analyzed according to genotype. **Results**: Paracetamol administration resulted in a significant reduction in pain scores at all time points (*p* < 0.001). Patients carrying the CYP2E15B variant exhibited significantly higher VAS scores and a weaker early analgesic response compared to non-carriers (*p* ≤ 0.001). Similarly, CYP2E11A carriers demonstrated higher pain scores across all time points (*p* < 0.05), although the magnitude of effect was less pronounced. No significant differences were observed for the CYP2E16 variant. Due to low frequency, CYP2E17B could not be reliably analyzed. **Conclusions**: Paracetamol is an effective analgesic in patients with lower extremity fractures; however, CYP2E1 polymorphisms may modulate individual pain perception and early analgesic response. In particular, the *5B and, to a lesser extent, *1A variants are associated with higher pain scores. These findings support the potential role of pharmacogenetic approaches in personalized pain management.

## 1. Introduction

Lower limb fractures, especially femur, tibia, and ankle fractures, are one of the most common injuries encountered in acute trauma practice and are usually accompanied by severe pain. Inadequate preoperative analgesia has been associated with increased sympathetic activation, hemodynamic fluctuations, stress response characterized by elevated cortisol, and catecholamine levels as well as development of chronic postoperative pain [1,2]. Therefore, implementation of effective and safe analgesic strategies during the preoperative period is of great clinical importance.

Paracetamol (acetaminophen) is a common analgesic, being a safe agent with reducing opioid requirement among others in a multimodal analgesic approach [3]. Intravenous form due to rapid onset of action and predictable pharmacokinetic profile, is the most preferred method in orthopedic trauma patients [4]. Although the exact mechanism of analgesic action of paracetamol is unknown, central cyclooxygenase inhibition, serotonergic pathway modulation, and endocannabinoid system-mediated effects have been reported to play a role in this [5]. Metabolism of paracetamol mostly occurs in the liver. While doing about 55–60% of the drug metabolized by glucuronidation and 30–35% sulfation, 5–10% portion undergoes oxidative metabolism by cytochrome P450 system [6]. Among the enzymes at this oxidative pathway, the one playing a key role is CYP2E1. Via CYP2E1, paracetamol is turned into a highly reactive toxic metabolite, N-acetyl-p-benzoquinone imine (NAPQI). NAPQI is normally detoxified by conjugation with hepatic glutathione; however, overproduction or depleted glutathione reserves may lead to hepatocellular damage [7].

Recent translational studies reveal that individual variations in CYP2E1 expression levels may influence the rate at which paracetamol is metabolized, thereby playing a role in both toxicity and pharmacodynamic response [8,9]. Different single nucleotide polymorphisms (SNPs) in the CYP2E1 gene that have been identified, may lead to changes in enzyme expression and activity. One of the most frequently studied variants is the RsaI/PstI polymorphism in the promoter region (c1/c2 alleles). The c2 allele has been reported in some populations to be associated with increased transcriptional activity [10]. Recent meta-analyses indicate that CYP2E1 polymorphisms can affect drug metabolism and risk of toxicity; however, the results vary depending on ethnic differences and sample size [11,12]. Although the role of CYP2E1 variations in paracetamol-induced hepatotoxicity has been studied, clinical data on its effect on analgesic efficacy are limited [13].

Pharmacogenetics research has evidenced that analgesic responses may significantly vary between individuals. Extensive studies have been carried out on CYP2D6 and UGT enzyme systems, which are involved in opioid metabolism [14]. In contrast, clinical studies on genetic markers for paracetamol are very limited. Theoretically, increased CYP2E1 activity could lead to a higher shift of paracetamol towards the oxidative metabolism pathway, thereby decreasing the concentration of the active analgesic in systemic circulation. Clinically, this may be reflected by lower reductions in VAS scores or even earlier loss of analgesic effect. Experimental models have demonstrated that induction of CYP2E1 increases the rate of paracetamol biotransformation [15].

Extremity fracture patients normally arrive in the hospital with extremely severe acute pain, and the risk of perioperative complications increases when effective analgesia is not provided during the early phase [1,2]. Furthermore, a high percentage of elderly population is found in this patient group and thus comorbidities and polypharmacy are quite common. Therefore, it is very important to provide analgesia, which is both effective and safe. If the CYP2E1 genotype affects the response to paracetamol, the standard dose administration may be insufficient in some patients, and this may lead to increased opioid requirements. Current perioperative guidelines underline the significance of personalized analgesia approaches [3,16].

Although the relationship between CYP2E1 polymorphisms and acetaminophen hepatotoxicity has been investigated in the literature, clinical studies that evaluate the relationship between analgesic efficacy and genetic variation in patients with acute orthopedic trauma are very limited. Therefore, exploring the association between the analgesic effect of preoperatively administered acetaminophen and CYP2E1 gene polymorphism in patients with lower extremity fracture not only will be a contribution to the pharmacogenetic literature but also forms the basis for personalized analgesic approaches. In this study, we aimed to evaluate the relationship between CYP2E1 genotype and changes in VAS scores following intravenous paracetamol administration in patients with lower extremity fractures. Although CYP2E1 polymorphisms have been mainly investigated in relation to paracetamol metabolism, hepatotoxicity, and toxic metabolite formation, their potential contribution to analgesic efficacy in acute orthopedic trauma remains poorly characterized. To the best of our knowledge, clinical evidence directly linking CYP2E1 variants with early VAS-based analgesic response after intravenous paracetamol administration in patients with lower extremity fractures is limited. Therefore, the novelty of the present study lies in evaluating CYP2E1-related variability not only as a metabolic or toxicity-related factor, but also as a potential determinant of short-term analgesic response in an acute fracture cohort.

## 2. Results

A total of 227 individuals were included in the study, comprising 127 patients with lower extremity fractures and 100 healthy controls. The mean age of the patient group was 61.26 ± 21.71 years. The sex distribution was balanced, with 66 females (52.0%) and 61 males (48.0%). No statistically significant differences were observed between the patient and control groups in terms of age and sex (all *p* > 0.05). The distribution of CYP2E1 genotypes in the patient and control groups is presented in Table 1. Genotype comparisons between patient and control groups were performed to evaluate population-level distribution differences, whereas clinical outcome analyses were restricted to the patient cohort. A statistically significant difference was observed in the distribution of CYP2E115B and CYP2E117B genotypes between the groups (*p* = 0.004 and *p* < 0.001, respectively). Specifically, the variant alleles of CYP2E1*5B and CYP2E1*7B were more frequently detected in the control group compared to the patient group. In contrast, no significant differences were found for CYP2E*1A and CYP2E1*6 genotypes (*p* > 0.05).

The analysis included 127 patients with lower extremity fractures, with a mean age of 61.26 ± 21.71 years and a balanced sex distribution. Pain intensity decreased progressively following paracetamol administration, as reflected by a reduction in mean VAS scores from 6.94 ± 1.09 at baseline to 5.98 ± 1.27 at 30 min, 5.08 ± 1.31 at 60 min, and 4.30 ± 1.33 at 120 min. Mutation-based analysis revealed that CYP2E1 polymorphisms influenced pain perception and analgesic response, with the CYP2E15B variant showing the strongest association, followed by CYP2E1*1A, while no significant effect was observed for CYP2E1*6. No homozygous mutant (mut/mut) genotypes were observed for any of the analyzed CYP2E1 polymorphisms. Hardy–Weinberg equilibrium was assessed using an exact test, and no significant deviation from HWE was observed for CYP2E1*1A, CYP2E1*5B, CYP2E1*6, or CYP2E1*7B (all *p* > 0.05).

Patients carrying the CYP2E1*5B variant exhibited significantly higher pain scores at all times compared to non-carriers (baseline: 7.56 vs. 6.82, *p* = 0.001; 30 min: 6.83 vs. 5.88, *p* < 0.001; 60 min: 5.88 vs. 4.92, *p* < 0.001; 120 min: 5.11 vs. 4.12, *p* < 0.001). Additionally, the reduction in VAS score between baseline and 30 min was significantly smaller in mutation carriers (0.73 vs. 0.94, *p* = 0.037), indicating a weaker early analgesic response (Table 2).

Carriers of the CYP2E1*1A variant also demonstrated higher VAS scores at all time points (baseline: 7.26 vs. 6.84, *p* = 0.035; 30 min: 6.49 vs. 5.90, *p* = 0.001; 60 min: 5.54 vs. 4.93, *p* = 0.001; 120 min: 4.65 vs. 4.17, *p* = 0.023). However, differences in VAS reduction (ΔVAS) were not statistically significant. For CYP2E1*6, no statistically significant differences were observed between mutation carriers and non-carriers at any time point (all *p* > 0.05) (Table 2).

Only three patients carried the CYP2E1*7B variant; therefore, no reliable statistical interpretation could be made for this polymorphism. Overall, the CYP2E1*5B polymorphism showed the strongest association with pain perception and reduced analgesic response, followed by CYP2E1*1A, whereas CYP2E1*6 showed no significant effect (Table 2).

**Figure 1:**
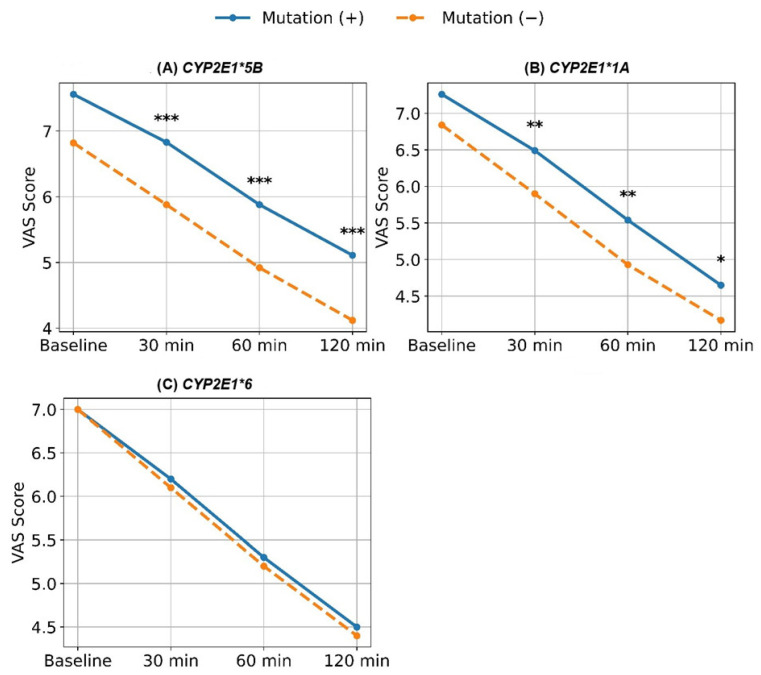
Comparison of pain scores (VAS) over time according to CYP2E1 polymorphisms. (**A**) Patients carrying the CYP2E1*5B variant exhibited significantly higher VAS scores at all time points compared to non-carriers, along with a reduced early analgesic response. (**B**) CYP2E1*1A carriers also demonstrated higher pain scores across all time points, although the effect was less pronounced. (**C**) No significant differences were observed between mutation carriers and non-carriers for CYP2E1*6. Data are presented as mean values. Statistical significance is indicated as * *p* < 0.05, ** *p* < 0.01, and *** *p* < 0.001.

Figure 1 illustrates the comparison of VAS scores over time according to CYP2E1 polymorphisms. Patients carrying the CYP2E1*5B variant exhibited significantly higher pain scores at all time points compared to non-carriers (all *p* ≤ 0.001), along with a reduced early analgesic response. Similarly, CYP2E1*1A carriers demonstrated higher VAS scores across all time points (*p* < 0.05), although the magnitude of the effect was less pronounced. In contrast, no significant differences were observed between mutation carriers and non-carriers for CYP2E1*6 (all *p* > 0.05).

**Figure 2:**
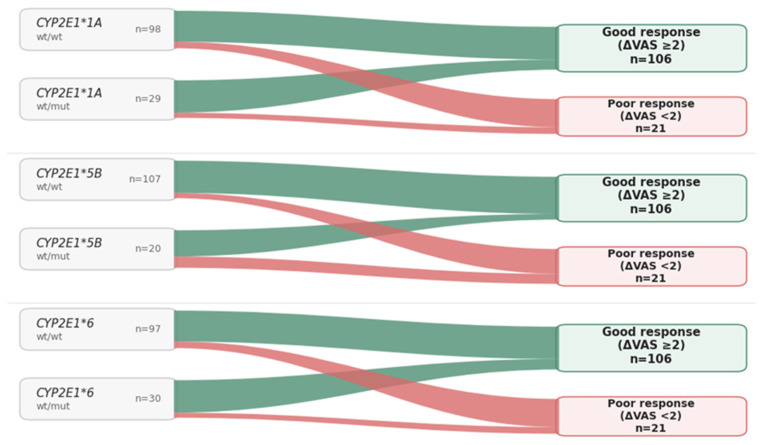
Sankey diagram illustrating the association between CYP2E1*1A, CYP2E1*5B, and CYP2E1*6 genotypes and analgesic response following paracetamol administration. Each polymorphism is presented separately to avoid double counting. Patients were classified as good responders (ΔVAS ≥ 2) or poor responders (ΔVAS < 2).

To further evaluate the relationship between CYP2E1 polymorphisms and analgesic response, patients were categorized as good responders (ΔVAS ≥ 2) and poor responders (ΔVAS < 2). A Sankey diagram was constructed to visualize the distribution of treatment response across genotype groups (Figure 2).

The Sankey diagram (Figure 2) provides a visual representation of the relationship between CYP2E1 genotypes and analgesic response. Patients were categorized as good responders (ΔVAS ≥ 2) and poor responders (ΔVAS < 2). To better characterize variability in analgesic response, patients were categorized as good responders (ΔVAS ≥ 2) and poor responders (ΔVAS < 2), allowing evaluation of response distribution beyond mean VAS values. The observed differences in responder proportions across genotype groups support the presence of interindividual variability in analgesic response. A higher proportion of poor responders was observed among carriers of the CYP2E1*5B variant compared to non-carriers, indicating a reduced analgesic response in this group. In contrast, non-carriers were more frequently represented among good responders. A similar but less pronounced pattern was observed for the CYP2E1*1A variant. No clear distribution pattern was identified for CYP2E1*6, consistent with the lack of significant association observed in statistical analyses.

## 3. Discussion

The main novelty of this study is that it evaluates CYP2E1 polymorphisms in relation to clinical analgesic response rather than focusing solely on paracetamol metabolism or hepatotoxicity. Previous studies have predominantly investigated CYP2E1 in the context of NAPQI formation, liver toxicity, and pharmacokinetic variability [6,7,9,10,11,12,17,18]. In addition, some studies have explored the influence of genetic polymorphisms on drug metabolism and plasma drug levels [13,19]. However, clinical evidence directly linking CYP2E1 genetic variation to analgesic response remains limited. In this context, the present study provides clinical evidence that CYP2E1*5B, and to a lesser extent CYP2E1*1A, may be associated with higher VAS scores and reduced early analgesic response after intravenous paracetamol administration in patients with lower extremity fractures.

This study is one of the few clinical research projects that evaluate the analgesic efficacy of intravenous paracetamol in patients with lower limb fracture and its relation to CYP2E1 gene polymorphisms [19,20,21]. The results indicate that paracetamol produced a significant reduction in pain in the entire patient group and CYP2E1 gene variants might have a modulating effect on the analgesic response [20,22,23,24]. The inclusion of a control group allowed comparison of genotype frequencies with a baseline population, strengthening the interpretation of the genetic findings.

In our study, significant reductions in VAS pain scores were observed at all time points following the administration of paracetamol (*p* < 0.001). This finding is consistent with previous studies that have demonstrated the efficacy of paracetamol in the management of acute pain. In fact, the literature highlights intravenous paracetamol as an effective analgesic in trauma and orthopedic surgery patients mainly due to its rapid onset of action and stable pharmacokinetic profile [3,4]. In addition, it is indicated as a safe option in multimodal analgesia approaches because of its opioid-sparing effect [16].

The analgesic effect of paracetamol is mainly explained by cyclooxygenase inhibition at the level of the central nervous system and modulation of the serotonergic system [5], while a major part of its metabolism occurs in the liver. Enzyme CYP2E1 converts a small fraction of the drug to toxic metabolite NAPQI during this metabolic process [7]. It has been previously shown that individual differences in CYP2E1 expression and activity can affect drug metabolism and subsequently clinical outcomes [9].

One of the most striking findings in our study was that individuals carrying the CYP2E15B variant had higher VAS scores at all the time points and early analgesic response was weaker. This led to the hypothesis that the variant that affects the promoter region might upregulate the enzyme expression and that indirect reduction in analgesic effect could be realized by increasing the oxidative metabolism of paracetamol. Similarly, higher pain scores were also found in CYP2E1*1A carriers, but it was seen that the effect was more limited. These findings lead to the thought that polymorphisms of the CYP2E1 gene may not only be involved in the toxicity but also in the pharmacodynamic response.

The major focus of researchers in the literature has been on the hepatotoxicity and drug metabolism potential of CYP2E1 polymorphisms. For instance, Fu et al. and Sharzehan et al. carried out meta-analyses, which revealed that CYP2E1 variants could change metabolic activity and disease risks [11,12]. However, clinical studies investigating the relationship between the analgesic effect of paracetamol and CYP2E1 genotype are very limited. Ulanova et al. (2024) [13] indicated that CYP2E1 polymorphisms could influence drug metabolite levels; hence, this may, in turn, affect clinical response. In this regard, our research is one of the significant pieces of evidence that directly relate CYP2E1 variants to analgesic response through clinical data [13]. Historically, the majority of research on CYP2E1 has focused on its role in hepatotoxicity, specifically the conversion of paracetamol into the toxic metabolite NAPQI [17,18]. High CYP2E1 activity, often seen in specific populations like those with morbid obesity, is linked to increased oxidation and potential safety risks [25].

There is a notable lack of clinical research directly connecting CYP2E1 polymorphisms to analgesic efficacy. While some small cohort studies have suggested non-significant associations between CYP2E1 promoter polymorphisms and paracetamol half-life, few have successfully established a direct link to the analgesic response in a clinical setting [26].

No significant difference was found in terms of CYP2E1*6 variant, which suggests that the functional effect of this polymorphism may be limited. Also, statistical analysis could not be done due to the low frequency of CYP2E1*7B variant. This situation is a common limitation of pharmacogenetic studies. It makes the evaluation of clinical effects of rare alleles difficult.

The variability in analgesic response among individuals is a well-known phenomenon from a pharmacogenetic perspective. The effects of CYP2D6 polymorphisms in opioid metabolism have been extensively studied [14,27]. On the other hand, the studies on genetic markers for non-opioid analgesics, like paracetamol, are quite limited. This research fills a major gap in the field by demonstrating that paracetamol response can also be genetically modulated.

Clinically, these findings reveal the need for personalized analgesia approaches. Considering the lower analgesic response in CYP2E1*5B carriers, multimodal analgesia strategies might be initiated earlier in these patients. Such a strategy might help prevent complications arising from inadequate pain control. Furthermore, pharmacogenetic test integration in clinical practice could enable individual analgesic planning.

Pain perception is inherently subjective and may be influenced by multiple factors, including fracture severity, individual pain thresholds, and psychological status. These variables were not systematically quantified in the present study and may act as potential confounders. In addition, pharmacokinetic parameters, such as plasma paracetamol levels and metabolite concentrations, were not measured. Therefore, the observed associations between CYP2E1 polymorphisms and analgesic response should be interpreted as associative rather than causal. Despite these limitations, the use of standardized VAS measurements at defined time points and a uniform analgesic protocol allowed for a controlled comparison of relative analgesic response across genotype groups. In the present study, this variability was partially addressed by categorizing patients into responder groups based on ΔVAS values, enabling evaluation beyond mean VAS scores. However, more advanced approaches, such as multivariable models adjusting for potential confounders, would provide a more comprehensive understanding of the relative contributions of genetic and non-genetic factors. This represents an important direction for future research.

This study has several limitations. First, it was conducted at a single center, and the sample size may not be sufficient to adequately assess rare polymorphisms. Second, only short-term analgesic response was evaluated, while long-term outcomes and additional analgesic requirements were not assessed. Furthermore, plasma levels of paracetamol and its metabolites were not measured, limiting the direct evaluation of the relationship between genotype and pharmacokinetics. In addition, liver biochemical parameters were not included, which may further restrict the interpretation of the pharmacogenetic effects of CYP2E1 polymorphisms. Another important limitation is the absence of homozygous mutant genotypes, which may reduce the statistical power to detect genotype–phenotype associations. Finally, haplotype or combined variant analyses were not performed. Although the combined effects of multiple CYP2E1 polymorphisms may better reflect enzymatic activity and clinical response, the relatively small sample size and lack of homozygous variants limited the feasibility and robustness of such analyses. Future studies with larger, multicenter cohorts incorporating pharmacokinetic, biochemical, and combined genetic analyses are warranted to provide a more comprehensive understanding of the role of CYP2E1 polymorphisms in analgesic response. In addition, the relatively small sample size for certain polymorphisms, particularly low-frequency variants such as CYP2E1*7B, may limit the statistical power and robustness of the findings. This limitation may reduce the ability to detect subtle genotype–phenotype associations and warrants cautious interpretation of the results.

More analyses should be conducted with larger patient groups, multicenter studies, and pharmacokinetic data, in future works. Besides being metabolized by CYP2E1, the four major pathways of paracetamol metabolism involve UGT and SULT, which contribute to forming non-toxic metabolites. Thus, evaluating these with CYP2E1 could be a step towards the more comprehensive understanding of paracetamol response. With this study, it is to be noted that paracetamol is an effective analgesic in patients with lower extremity fracture. Moreover, CYP2E1 gene polymorphisms might have modulator roles on the analgesic response. The association of CYP2E1*5B and to a certain extent *1A variants with higher pain scores, particularly, highlight the importance of pharmacogenetic-based personalized analgesia approaches. Our results provide a significant groundwork for the use of genetic data in clinical practice.

## 4. Materials and Methods

### 4.1. Study Design

The present study was designed as a prospective observational case–control study. Between November 2023 and October 2024, this study was conducted at Pamukkale University Faculty of Medicine Department of Orthopedics and Traumatology. Among patients who were admitted to emergency room with lower extremity fractures due to trauma and scheduled for surgery, 120 patients who had preoperative pain complaints were included in the study. In addition, a control group comprising 100 healthy individuals was also included in the study for comparison purposes. The healthy control group was included solely for comparison of CYP2E1 genotype distributions at the population level. Pain assessment and analgesic response analyses were performed exclusively in the patient group. The patients who constituted the study group were evaluated according to the determined inclusion and exclusion criteria. The present study was a prospective case–control study, and the required approval was obtained from the Ethics Committee of Pamukkale University prior to the study (No. E-60116787-020-434980, date: 13 October 2023).

### 4.2. Inclusion Criteria Were Defined Separately for the Patient and Control Groups

Patients aged 18 years and older presenting with lower extremity fractures and preoperative pain complaints were included in the patient group. The control group consisted of healthy adult individuals aged 18 years and older.

Exclusion criteria for the patient group included: use of any analgesic medication within the last 6 h; chronic renal failure, liver cirrhosis, pregnancy, known hypersensitivity to paracetamol or any of its components, use of monoamine oxidase inhibitors within the previous 14 days, treatment-resistant epilepsy, and refusal to provide informed consent.

Although recent analgesic use was controlled by the 6 h exclusion criterion, detailed information regarding chronic pain conditions or long-term use of centrally acting medications (such as pregabalin or opioids) was not systematically recorded.

### 4.3. Drug Administration and Pain Assessment

**Figure 3:**
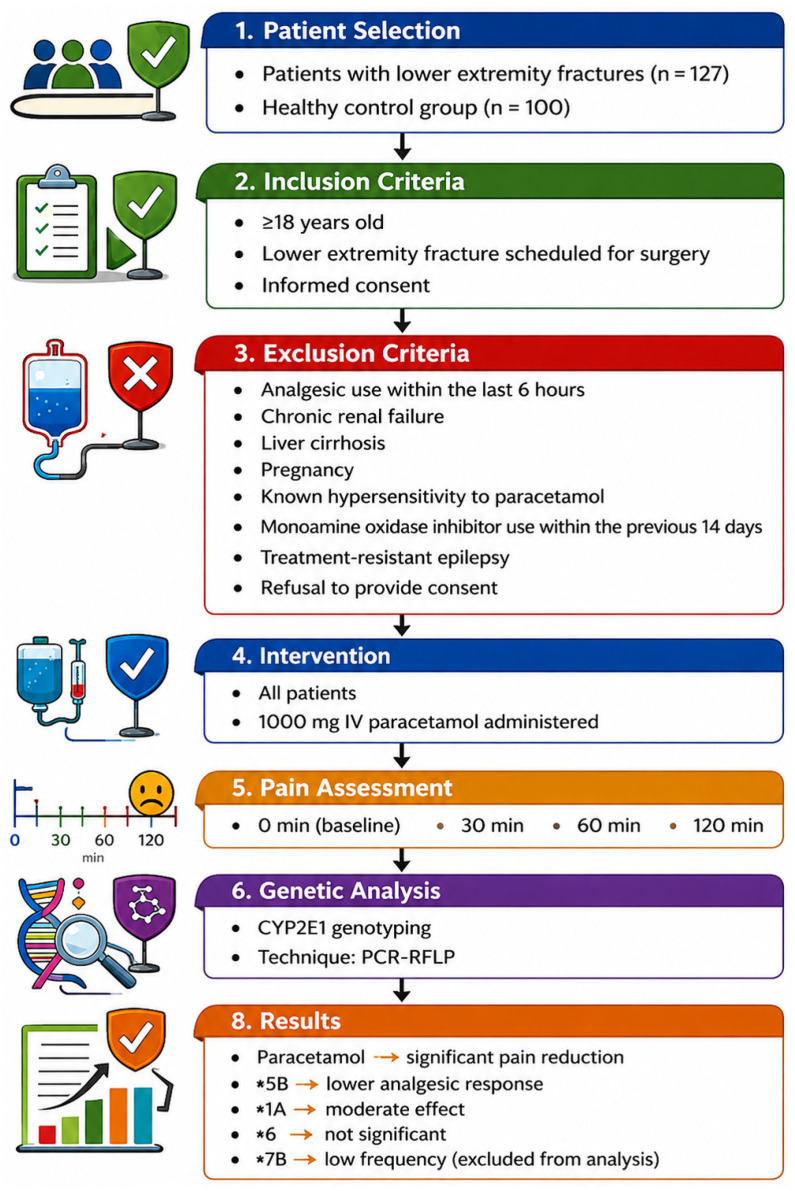
Study flowchart showing patient selection, grouping, and analyses performed.

In the study, analgesic therapy was the use of paracetamol (Parol IV). Paracetamol was administered as a single dose of 1000 mg intravenous infusion (in 100 mL solution) over 15–20 min. Pain levels of patients were evaluated by using VAS. Pain assessment was performed at 0, 30, and 60 min and 2 h. Also, patients were followed up for vital signs and possible side effects. At the end of the 2nd hour, the study was terminated. If the drug-related side effects were detected during the study, the study was terminated immediately. Among the possible side effects, local reactions to the injection site, redness of the skin, rash, itching, flushing of the face, hives, heartburn, stomachache, nausea, vomiting, headache, dizziness, erythema, and urticaria were listed. Figure 3 illustrates the flow of participants and the analysis sets. ΔVAS was calculated as the difference between baseline VAS score and the VAS score at each subsequent time point (ΔVAS = VAS_baseline − VAS_timepoint). This calculation was performed separately for 30, 60, and 120 min following paracetamol administration.

### 4.4. Visual Analog Scale

Visual Analog Scale (VAS) is a technique by which some parameters that are difficult to quantify numerically can be quantitatively evaluated, and it is a method widely used for measuring pain severity. This scale is based on the individual’s marking their present state on a 100 mm-long line whose ends are defined. VAS is often scoring on a scale from 0 to 10. In this system, 0 point refers to the absence of pain, whereas 10 point indicates pain of unbearable intensity. The intensity of pain is classified as mild pain for 0 to 3 points, moderate pain for 3 to 6 points, and severe pain for 6 points and above. In this study, the patients were requested to rate their pain intensities in the preoperative period on a scale of 0 (no pain at all) to 10 (the most severe pain). This scoring system enables the transformation of patients’ subjective pain experiences into objective and comparable data, thus allowing a quantitative evaluation of treatment effectiveness [28].

### 4.5. Blood Samples and DNA Isolation

Before Parol application, 2 mL of venous blood was collected from the patients and placed into vacuum tubes containing K3EDTA. Genomic DNA was isolated from the blood samples obtained from individuals in patient and control groups by using a commercial DNA isolation kit according to the manufacturer’s instructions.

### 4.6. CYP2E1 Genotyping

Genotyping of CYP2E1 gene polymorphisms were carried out using the polymerase chain reaction–restriction fragment length polymorphism (PCR-RFLP) method. For CYP2E1*5B polymorphism, the fragment of approximately 410–413 bp corresponding to the 5′-regulatory regions of the gene was amplified by using forward 5′-CCAGTCGAGTCTACATTGTCA-3′ and reverse 5′-TTCATTCTGTCTTCTAACTGG-3′ primers. PCR amplification was done with 35 cycles of denaturation at 94 °C for 1 min, annealing at 55 °C for 1 min, and extension at 72 °C for 1 min after the initial denaturation, followed by a final extension at 72 °C for 6 min. The PCR products obtained were digested with the RsaI restriction enzyme at 37 °C and fragments approximately 352 and 61 bp in length were assessed through agarose gel electrophoresis. Forward 5′-TCGTCAGTTCCTGAAAGCAGG-3′ and reverse 5′-GAGCTCTGATGCAAGTATCGCA-3′ primers were used in PCR amplification for CYP2E1*6 polymorphism. PCR conditions were as follows: 5 min initial denaturation at 94 °C, then 35 cycles of 1 min denaturation at 94 °C, 1 min annealing at 61 °C and 1 min extension at 72 °C, and finally a last extension of 6 min at 72 °C. The amplified PCR products were analyzed following restriction digestion with DraI. In the case of CYP2E1*7B polymorphism, a fragment of about 360 bp from the gene’s promoter region was amplified using forward 5′-GTGGCTGGAGTTCCCCGTTG-3′ and reverse 5′-TGCTGCCAGCCCGGGAGGAC-3′ primers. PCR conditions were performed for 5 min initial denaturation at 94 °C, then 30 cycles of 1 min denaturation at 94 °C, 1.5 min annealing at 62 °C and 2 min extension at 72 °C, and finally 10 min last extension at 72 °C. PCR products were digested with the DdeI restriction enzyme. The analyzed CYP2E1 polymorphisms included *5B (rs2031920), *6 (rs6413432), and *7B (rs6413420).

### 4.7. Statistical Analysis

Statistical analyses were performed using the IBM SPSS for Windows Version 25.0 (IBM Corp., Armonk, NY, USA) software package. Descriptive statistics of data were given as frequency (*n*) and percentage (%) for categorical variables, whereas continuous variables mean ± standard deviation was given, along with median, minimum, and maximum values. Normality of continuous variables was checked through Kolmogorov–Smirnov and Shapiro–Wilk tests. Independent samples *t*-test (Student’s *t*-test) was used for the comparison of continuous variables that showed normal distribution between two independent groups. Mann–Whitney U test was applied for the comparison of the data that did not meet the normal distribution assumption. Wilcoxon signed-rank test was used for the comparison of pre- and post-Parol application measurements. Pearson Chi-square test was used for the evaluation of the relationship between categorical variables. In all statistical analyses, the value of *p* < 0.05 was accepted as statistically meaningful.

## Figures and Tables

**Table 1:** Distribution of CYP2E1 genotypes in patient and control groups.

Genotype	Patient Group *n* (%)	Control Group *n* (%)	*p*-Value	HWE (p)
CYP2E1*A **wt/wt**	98 (77.2)	87 (87.0)	0.081	0.367
CYP2E1*A **wt/mut**	29 (22.8)	13 (13.0)		
CYP2E1*5B **wt/wt**	107 (84.3)	67 (67.0)	0.004	1.000
CYP2E1*5B **wt/mut**	20 (15.7)	33 (33.0)		
CYP2E1*6 **wt/wt**	97 (76.4)	83 (83.0)	0.215	0.214
CYP2E1*6 **wt/mut**	30 (23.6)	17 (17.0)		
CYP2E1*7B **wt/wt**	124 (97.6)	78 (78.0)	<0.001	1.000
CYP2E1*7B **wt/mut**	3 (2.4)	22 (22.0)		

**Table 2:** Comparison of VAS scores according to CYP2E1 mutations.

Mutation	Time Point	Mutation (+) Mean ± SD	Mutation (−) Mean ± SD	*p*-Value
*1A	Baseline	7.26 ± (0.81)	6.84 ± (0.87)	0.035
	30 min	6.49 ± (0.81)	5.90 ± (0.85)	0.001
	60 min	5.54 ± (0.77)	4.93 ± (0.89)	0.001
	120 min	4.65 ± (0.84)	4.17 ± (0.98)	0.023
*5B	Baseline	7.56 ± (0.75)	6.82 ± (0.84)	0.001
	30 min	6.83 ± (0.71)	5.88 ± (0.82)	<0.001
	60 min	5.88 ± (0.79)	4.92 ± (0.83)	<0.001
	120 min	5.11 ± (0.97)	4.12 ± (0.89)	<0.001
*6	All	No significant difference		>0.05
*7B	All	Not analyzed (*n* = 3)		—

## Data Availability

The original contributions presented in this study are included in the article. Further inquiries can be directed to the corresponding authors.

## Abbreviations

The following abbreviations are used in this manuscript:

CYP2E1	cytochrome P450 2E1
NAPQI	N-acetyl-p-benzoquinone imine
VAS	Visual Analog Scale
PCR-RFLP	polymerase chain reaction–restriction fragment length polymorphism
